# Unraveling the mitochondrial genome of the medicinal Chinese motherwort (*Leonurus japonicus*, Lamiaceae): structural dynamics, organelle-to-nuclear gene transfer, and evolutionary implications

**DOI:** 10.3389/fpls.2025.1546449

**Published:** 2025-06-03

**Authors:** Xinyu Bai, Tingting Zhu, Huiru Chen, Xiaoqun Wang, Jing Liu, Yuqing Feng, Yanbo Huang, Joongku Lee, Goro Kokubugata, Zhechen Qi, Xiaoling Yan

**Affiliations:** ^1^ Zhejiang Province Key Laboratory of Plant Secondary Metabolism and Regulation, College of Life Sciences and Medicine, Zhejiang Sci-Tech University, Hangzhou, China; ^2^ Eastern China Conservation Centre for Wild Endangered Plant Resources, Shanghai Chenshan Botanical Garden, Shanghai, China; ^3^ Department of Environment and Forest Resources, Chungnam National University, Daejeon, Republic of Korea; ^4^ Department of Botany, National Museum of Nature and Science, Tsukuba, Ibaraki, Japan

**Keywords:** *Leonurus japonicus*, mitochondrial genome, repeat sequence, synteny analysis, RNA editing, long-read sequencing, phylogenetic relationships, plastid-derived DNA

## Abstract

**Introduction:**

*Leonurus japonicus* (Chinese motherwort) is a medicinal Lamiaceae species renowned for its pharmacological compounds, yet its mitochondrial genome remains unexplored. Elucidating mitogenomic structure and evolution can inform plant genetics, phylogenetics, and molecular breeding.

**Methods:**

We assembled the complete mitochondrial genome of *L. japonicus* using a combination of Oxford Nanopore long reads and Illumina short reads. Three assembly strategies—*de novo* assembly with PMAT and Flye, and hybrid assembly with Unicycler—were integrated and validated via read mapping and comparison to reference mitogenomes (*Salvia miltiorrhiza, Arabidopsis thaliana, Liriodendron tulipifera*). Annotation employed GeSeq, tRNAscan-SE, and manual curation. Repeat elements (SSR, tandem, dispersed) were identified with MISA, TRF, and REPuter; plastid‐to‐mitochondrion transfers (MTPTs) were detected by BLASTN against the assembled plastome; and RNA editing sites were predicted using Deepred-mt. Phylogenetic and synteny analyses were conducted with IQ-TREE, MAFFT alignments of 24 conserved PCGs, and NGenomeSyn visualization.

**Results:**

The circular mitogenome spanned 384,199 bp (45.1% GC) and encoded 35 protein-coding genes, 11 tRNAs, and 3 rRNAs. We detected 241 SSRs, 13 tandem repeats, and 90 dispersed repeats, indicating extensive recombination potential. Thirty-one MTPTs totaling 24,818 bp (6.46% of the mitogenome) were identified. Comparative analyses revealed strong purifying selection (Ka/Ks < 1) across most PCGs, with selective signatures in atp4 and ccmB. Phylogenetic inference placed *L. japonicus* among Lamiales, closely allied to *Scutellaria tsinyunensis* and *Rotheca serrata*. Synteny maps demonstrated frequent genome rearrangements. Deepred-mt predicted 408 C-to-U RNA editing sites, notably in nad4 and *ccmB*, including novel start and stop codons.

**Discussion:**

The *L. japonicus* mitogenome exhibits marked structural plasticity, reflecting dynamic repeats and organelle‐to‐organelle DNA transfers. Extensive RNA editing underscores post-transcriptional regulation in mitochondrial function. These findings enrich genomic resources for *Leonurus*, support phylogenetic and evolutionary studies in Lamiaceae, and lay groundwork for molecular breeding and conservation strategies targeting mitochondrial traits.

## Introduction

The mint family (Lamiaceae) is the sixth-largest family of angiosperms, comprising over 7,000 species across 236 genera. Many species are of considerable cultural and economic importance, serving as key resources in agriculture, food, and medicine (Boachon et al., 2018; Uritu et al., 2018; Michel et al., 2020; Sedano-Partida et al., 2020; Tjitraresmi et al., 2020; Rodríguez-López et al., 2022). Lamiaceae is particularly renowned for its remarkable diversity of secondary metabolites, especially terpenoids and phenolic compounds, which possess a wide range of biological activities and have been widely applied in aromatherapy, food preservation, and pharmaceuticals (Wu et al., 2012; Boachon et al., 2018; Tjitraresmi et al., 2020; Rodríguez-López et al., 2022; Moshari-Nasirkandi et al., 2023; Wang et al., 2024a). Distinct from the terpenoid- and phenolic-rich members of the family, the genus *Leonurus* (motherwort), exhibits prominent medicinal activity through a different class of compounds—alkaloids, including stachydrine and leonurine (Fierascu et al., 2019; Li et al., 2024). These bioactive compounds are particularly noted for their potential in treating cardiovascular conditions and gynecological disorders, positioning *Leonurus* as a promising candidate for therapeutic development in traditional and modern medicine.

*Leonurus japonicus* Houtt., commonly known as Chinese motherwort or “Yi Mu Cao,” is native to Asia and has been used for centuries in Traditional Chinese Medicine to treat a variety of obstetrical and gynecological conditions (Shang et al., 2014; Miao et al., 2019). The aerial parts of *L. japonicus* have traditionally been used to treat irregular menstruation, dysmenorrhea, amenorrhea, blood stasis, postpartum hemorrhage, edema, and oliguria (Ahmed et al., 2006; Shang et al., 2014; Commission, C. P and Others, 2015; Miao et al., 2019; Fierascu et al., 2019; Wang et al., 2023). As a result, *L. japonicus* is listed among the 50 fundamental herbs in the Chinese materia medica (Shang et al., 2014). Given its well-established role in traditional medicine and expanding use in modern healthcare applications, *L. japonicus* represents not only a medicinally significant species but also one with considerable economic potential. Contemporary pharmacological research has demonstrated that the active compounds in *L. japonicus* exhibit cardioprotective, antioxidative, and neuroprotective effects, contributing to its growing popularity in women’s health products and cosmetics (Shang et al., 2014).

To support the broader development and utilization of this species, a more comprehensive understanding of its genetic and molecular basis is essential. Recent studies have explored the anti-inflammatory properties and disease resistance mechanisms of *L. japonicus* (Fierascu et al., 2019; Miao et al., 2019), along with investigations into the biosynthesis and metabolic pathways of its bioactive compounds (Li et al., 2024). However, despite these advances, challenges related to cultivation persists, particularly in terms of yield stability and quality control. Like many long-domesticated medicinal plants, *L. japonicus* suffers from reduced genetic diversity due to the founder effects and hybridization of germplasms, resulting in diminished genetic diversity (Peng et al., 2014; Wang et al., 2022a). Recent population genetic studies have reported a relatively low nucleotide diversity index (*Pi* = 0.00029) among wild and cultivated *L. japonicus* accessions (Wang et al., 2023). Addressing these limitations through genetic improvement and resource conservation is critical for enhancing agronomic traits and unlocking the species’ full pharmacological and economic potential.

While nuclear genome resources for *Leonurus* have recently become available and have facilitated advances in leonurine biosynthesis research (Li et al., 2024), studies on organellar genomes, particularly mitochondrial genomes (mitogenomes), which remain limited. Although often overlooked in plant genomic studies (Daniell et al., 2016; Wang et al., 2024b), organellar genomes play essential roles in phylogenetic inference (Gitzendanner et al., 2018; Li et al., 2021a; Zhao et al., 2021; Yin et al., 2022; Cao et al., 2023) and species identification, especially in medicinal plants (Chai et al., 2022; Wang et al., 2022b; Xu et al., 2022; You et al., 2022; Guo et al., 2023; Li et al., 2023). Within Lamiaceae, plastid genomes have been relatively well studied (Tao et al., 2022; Yin et al., 2022; Cheng et al., 2023), yet complete mitogenomes have only been reported for a few genera, including *Ajuga* (Zhu et al., 2014), *Erythranthe*, *Rotheca*, *Salvia* (Chen and Liu, 2019), and *Scutellaria* (Li et al., 2021b), representing less than 2% of the genera in the family.

Despite the limited number of studies on mitogenomes in Lamiaceae, their importance in plant biology is indisputable. Mitochondria are indispensable for plant growth and development, participating in a wide range of essential cellular functions, including ATP production, energy metabolism, photorespiration, amino acid and coenzyme biosynthesis, and programmed cell death (Ogihara et al., 2005; Maréchal and Brisson, 2010; Daniell et al., 2016; Wang et al., 2024b). They also play a crucial role in abiotic stress responses by facilitating adaptations to drought, regulating respiration under salt stress, enhancing heat tolerance, and mediating flood responses through complex metabolic networks (Pastore et al., 2007; Jacoby et al., 2011; Mustafa and Komatsu, 2016). Moreover, structural recombination in plant mitogenomes is closely linked to cytoplasmic male sterility (CMS), a key trait in hybrid breeding programs. These characteristics underscore the value of mitochondria in genetic engineering, plant breeding, and the development of biological vaccines (Daniell et al., 2016; Tao et al., 2022; Wang et al., 2024b). The emergence of advanced mitochondrial genome-editing tools—such as mitoTALENs (Kazama et al., 2019; Arimura et al., 2020; Takatsuka et al., 2022), the Golden Gate cloning system (Kang et al., 2021), and TALEN-GDM (Forner et al., 2022)—has opened new avenues for introducing stable, inheritable mutations in plant mitogenomes (Maliga, 2022). These advances are particularly promising given the strong DNA repair mechanisms and low mutation rates of plant mitochondria, which ensure the long-term stability of engineered traits (Christensen, 2013; Kazama et al., 2019).

Structurally, plant mitogenomes are markedly more dynamic and variable than their mammalian counterparts. Although initially described as circular “master circles” (Kubo et al., 2000; Notsu et al., 2002; Handa, 2003; Ogihara et al., 2005), recent advances have revealed a much more dynamic structure. Long-read sequencing has shown that many plant mitogenomes exist as multipartite or branched linear forms, resulting from frequent recombination events mediated by large repeat sequences (Maréchal and Brisson, 2010; Sloan, 2013; Kozik et al., 2019). These structural variations are largely regulated by nuclear genes, especially the MutS homolog 1 (*MSH1*), which suppresses ectopic recombination. Loss-of-function mutations in *MSH1* lead to elevated recombination frequency and extensive genomic rearrangements (Zou et al., 2022; Zhu et al., 2025), highlighting the key role of nuclear-cytoplasmic interaction in maintaining mitogenomic integrity (Wu et al., 2020; Zou et al., 2022). Further evidence from recent studies in cotton (Kong et al., 2025) and *Arabidopsis* (Zhu et al., 2025) reinforces the idea that plant mitogenome evolution is shaped not only by intrinsic sequence features but also by the regulatory landscape imposed by nuclear-encoded factors.

Paradoxically, despite their structural plasticity, plant mitogenomes evolve slowly at the sequence level, displaying remarkably low point mutation rates (Ghulam et al., 2015; Møller et al., 2021). Several mechanisms may contribute to this stability, including efficient homologous recombination-mediated repair and high genome copy numbers (Fan et al., 2022; Wang et al., 2024c; Zwonitzer et al., 2024). For example, Fan et al. (2022) demonstrated that in *Fragaria*, despite extensive structural variation, the sequence divergence remains low due to efficient repair via microinversion-associated recombination. Similarly, Zwonitzer et al. (2024) found that mitogenomes with higher copy numbers tend to exhibit slower evolutionary rates, further supporting the protective role of genome redundancy. These observations suggest that plant mitochondria have evolved a complex regulatory system that simultaneously permits structural remodeling while maintaining functional and sequence stability, which may be a key adaptive strategy in long-lived or stress-exposed species (Nguyen et al., 2020).

Given the medicinal and economic importance of *L. japonicus* and the lack of mitogenomic data in the genus, we present here a comprehensive analysis of its mitogenome. This study focuses on genome assembly, repeat structure, RNA editing, and phylogenetic placement within Lamiales, alongside comparative analysis with its plastid and nuclear genomes. These findings are expected to enhance our understanding of mitogenome evolution in Lamiaceae and support future efforts in species conservation and molecular breeding.

## Materials and methods

### DNA extraction and mitogenome assembly

*L. japonicus* specimens were collected from Mt. Emei, Sichuan Province, and cultivated at Shanghai Chenshan Botanical Garden. High-quality genomic DNA was extracted from stem epidermal tissue using a modified CTAB protocol (Arseneau et al., 2017). Sequencing was performed on both Illumina and Oxford Nanopore platforms by Wuhan Benagen Tech Solutions Company (http://en.benagen.com). For short-read sequencing, Illumina HiSeq Xten PE150 generated 12.5 Gb of clean data (2×150 bp paired-end reads), providing approximately 50× genome coverage after quality control. Long-read sequencing was performed on the GridION X5 platform (Oxford Nanopore Technologies, Oxford, UK), yielding ~1,225,586 reads with a total length of 20.75 Gb. These reads had a maximum length of 212,466 bp and an average length of ~16,928 bp. Raw Nanopore reads were then corrected using NextDenovo v2.5.1 (Hu et al., 2024). To accurately assemble the mitogenome of *L. japonicus*, we employed three independent strategies: 1) PMAT v1.5.3 (Bi et al., 2024) for *de novo* assembly of corrected Nanopore reads (~4.33 Gb). 2) Flye v2.9.2 (Kolmogorov et al., 2019) for independent assembly, with Bandage v0.8.1 (Wick et al., 2015). 3) a hybrid approach using Unicycler v0.4.8 (Wick et al., 2017), combining corrected Nanopore long reads and Illumina short reads (assembled via SPAdes v3.15.4 (Bankevich et al., 2012)), with repeat regions resolved using minimap2 v2.28 (Li, 2018). Conserved mitochondrial genes from *Salvia miltiorrhiza* (NC023209), *Arabidopsis thaliana* (NC037304), and *Liriodendron tulipifera* (NC021152) were used as BLASTn queries to validate mitochondrial contigs. Read mapping was performed using BWA v0.7.17 (Li, 2013) to assess assembly consistency. The final genome structure was determined by integrating and comparing results from all three methods.

### Annotation of the mitogenome

The mitogenome was annotated using GeSeq v2.03 (Tillich et al., 2017) with three reference mitogenomes mentioned above. *Salvia miltiorrhiza* was selected for its close phylogenetic relationship to *L. japonicus*, *Arabidopsis thaliana* for its high annotation accuracy, and *Liriodendron tulipifera* for its inclusion of a complete set of 24 core and 17 variable mitochondrial genes. Transfer RNAs (tRNAs) were identified using tRNAscan-SE v.2.0.11 (Chan et al., 2021), while rRNAs were annotated using BLASTN. Any annotation errors in the mitogenome were manually corrected using Apollo v1.11.8 (Lewis et al., 2002).

### Selective pressure analysis

To investigate the selective pressure and potential adaptive evolution of conserved mitochondrial protein-coding genes (PCGs) in *L. japonicus* and its close relatives, we conducted a comparative Ka/Ks analysis. GenBank files of the target species were retrieved from the NCBI database, and both coding sequences (CDS) and corresponding protein sequences were extracted. Homologous gene pairs were identified by performing BLASTP searches of each species’ protein sequences against the *L. japonicus* reference proteome to identify the best-matching orthologs. The identified orthologous protein sequences were aligned using MAFFT (Katoh and Standley, 2013), and the resulting alignments were used to guide codon-based alignments of the corresponding nucleotide sequences with a custom Perl script. Finally, KaKs_Calculator v3.0 (Zhang, 2022) was employed to compute the nonsynonymous (Ka) to synonymous (Ks) substitution rate ratio (Ka/Ks) for each orthologous gene pair, providing insights into the evolutionary selection acting on mitochondrial genes.

### Relative synonymous codon usage

Relative synonymous codon usage (RSCU) analysis was performed by extracting the protein-coding sequences from the genome using Phylosuite v1.1.16 (Zhang et al., 2020). The protein-coding genes of the mitogenome were then subjected to codon preference analysis using MEGA v7.0, and the RSCU values were calculated (Kumar et al., 2016). An RSCU value>1 indicates that the codon is preferentially used by amino acids, whereas an RSCU value<1 indicates the opposite trend.

### Analysis of repeat elements

We identified simple sequence repeats (SSRs) using the online website MISA (https://webblast.ipk-gatersleben.de/misa/) (Beier et al., 2017), and tandem repeats were identified using TRF v4.09 (https://tandem.bu.edu/trf/trf.unix.help.html) (Benson, 1999). In addition, forward, reverse, palindromic, and complementary repeat sequences were identified using REPuter (https://bibiserv.cebitec.uni-bielefeld.de/reputer/) (Kurtz et al., 2001) with the following settings: hamming distance of three, maximum computed repeats of 5000 and minimal repeat size of 30 bp. The results were visualized using the Circos package v0.69.9 (Zhang et al., 2013).

### Phylogenetic analysis

To assess the phylogenetic position of *L. japonicus* within the asterid clade, we selected representative species from the orders Lamiales, Solanales, Ericales, and Asterales based on the APG system. A total of 15 species from Lamiales were included, along with four from Solanales, two from Ericales, and two from Asterales. The Asterales species served as outgroups due to their suitable phylogenetic distance from the target taxa. The selected Lamiales species were *Ajuga ciliata*, *A. reptans*, *Castilleja paramensis*, *Dolichandrone* sp*athacea*, *Dorcoceras hygrometricum*, *Erythranthe guttata*, *Haberlea rhodopensis*, *Hesperelaea palmeri*, *Ligustrum quihoui*, *Markhamia stipulata* var. *kerrii*, *Osmanthus fragrans*, *Rotheca serrata*, *Salvia miltiorrhiza*, *Scutellaria tsinyunensis*, and *Utricularia reniformis*. The Solanales species included *Nicotiana sylvestris*, *Nicotiana tabacum*, *Solanum lycopersicum*, and *Solanum melongena*. The Ericales species were: *Camellia sinensis* and *Vaccinium macrocarpon*. The Asterales outgroup species were *Codonopsis lanceolata* and *Helianthus annuus*. A set of 24 conserved mitochondrial protein-coding genes (*atp*1, *atp*4, *atp*6, *atp*8, *atp*9, *ccm*B, *ccm*C, *ccm*FC, *ccm*FN, *cob*, *cox*2, *cox*3, *mat*R, *nad*1, *nad*2, *nad*3, *nad*4L, *nad*5, *nad*6, *nad*7, *nad*9, *rpl*5, *rps*3, and *rps*12) were extracted using PhyloSuite (Zhang et al., 2020) and aligned with MAFFT v7.505 (Katoh and Standley, 2013) for multiple sequence comparisons. Phylogenetic analysis was performed using IQ-TREE v2 (Minh et al., 2020), and the resulting maximum likelihood (ML) tree was visualized with iTOL v4 (Letunic and Bork, 2019). These genes are highly conserved across angiosperm mitogenomes and span multiple functional categories.

### Synteny and intracellular gene transfer analysis

To investigate mitogenome variation and recombination among *L. japonicus* and closely related species, we conducted a synteny analysis involving the mitogenomes of *L. japonicus*, *Scutellaria tsinyunensis*, *Rotheca serrata*, *Ajuga reptans*, *A. ciliata*, *Salvia miltiorrhiza*, *Castilleja paramensis*, and *Erythranthe guttata*. Based on phylogenetic proximity, pairwise BLASTN searches were performed among these mitogenomes. Homologous sequences ≥500 bp were retained as conserved collinear blocks. These blocks were visualized using NGenomeSyn (He et al., 2023) to generate multi-genome synteny maps.

Mitochondrial plastid DNAs (MTPTs) are plastid-derived DNA fragments integrated into the mitogenome. These sequences provide valuable insights into historical DNA transfer events between organelles and play a potential role in mitogenome evolution and function. To identify MTPTs in *L. japonicus*, we first assembled the plastid genome using GetOrganelle v1.7.5 (Jin et al., 2020), followed by annotation with CPGAVAS2 (Shi et al., 2019) and manual correction using CPGView (Liu et al., 2023). The assembled plastome was then compared to the published plastome sequence of *L. japonicus* (Zhang et al., 2018) to validate assembly accuracy. BLASTN searches were then performed between the plastome and mitogenome using the following parameters: *E-value* = 1e-5, *NumofHits* = 50,000, and *NumofAligns* = 25,000. The BLAST results were visualized and analyzed using TBtools (Chen et al., 2020) to identify homologous regions indicative of MTPTs.

In addition, to identify potential homologous sequences transferred between the organellar and nuclear genomes of *L. japonicus*, the complete nuclear genome (GCA030762865.1) was downloaded from NCBI (https://www.ncbi.nlm.nih.gov/datasets/genome/GCA_030762865.1/). BLASTN searches were then independently conducted between the plastid and mitogenomes and the nuclear genome. The resulting alignments were analyzed to detect homologous fragments corresponding to genes encoded in the plastome and mitogenome, thereby determining their locations on the nuclear chromosomes. Finally, the R package RIdeogram (Hao et al., 2020) was used to visualize the chromosomal distribution of these homologous fragments and associated genes, illustrating potential intracellular gene transfer events in *L. japonicus*.

### RNA editing site prediction

To identify potential C-to-U RNA editing sites in the mitochondrial PCGs of *L. japonicus*, we analyzed all annotated PCG sequences. Predictions were performed using Deepred-mt (Edera et al., 2021), a convolutional neural network-based tool specifically designed for plant mitochondrial RNA editing site identification. This method offers improved prediction accuracy compared to traditional rule-based approaches. Only editing sites with a predicted probability score >0.9 were retained to ensure high confidence in the results.

## Results

### Assembly and annotation of the mitogenome

**Figure 1:**
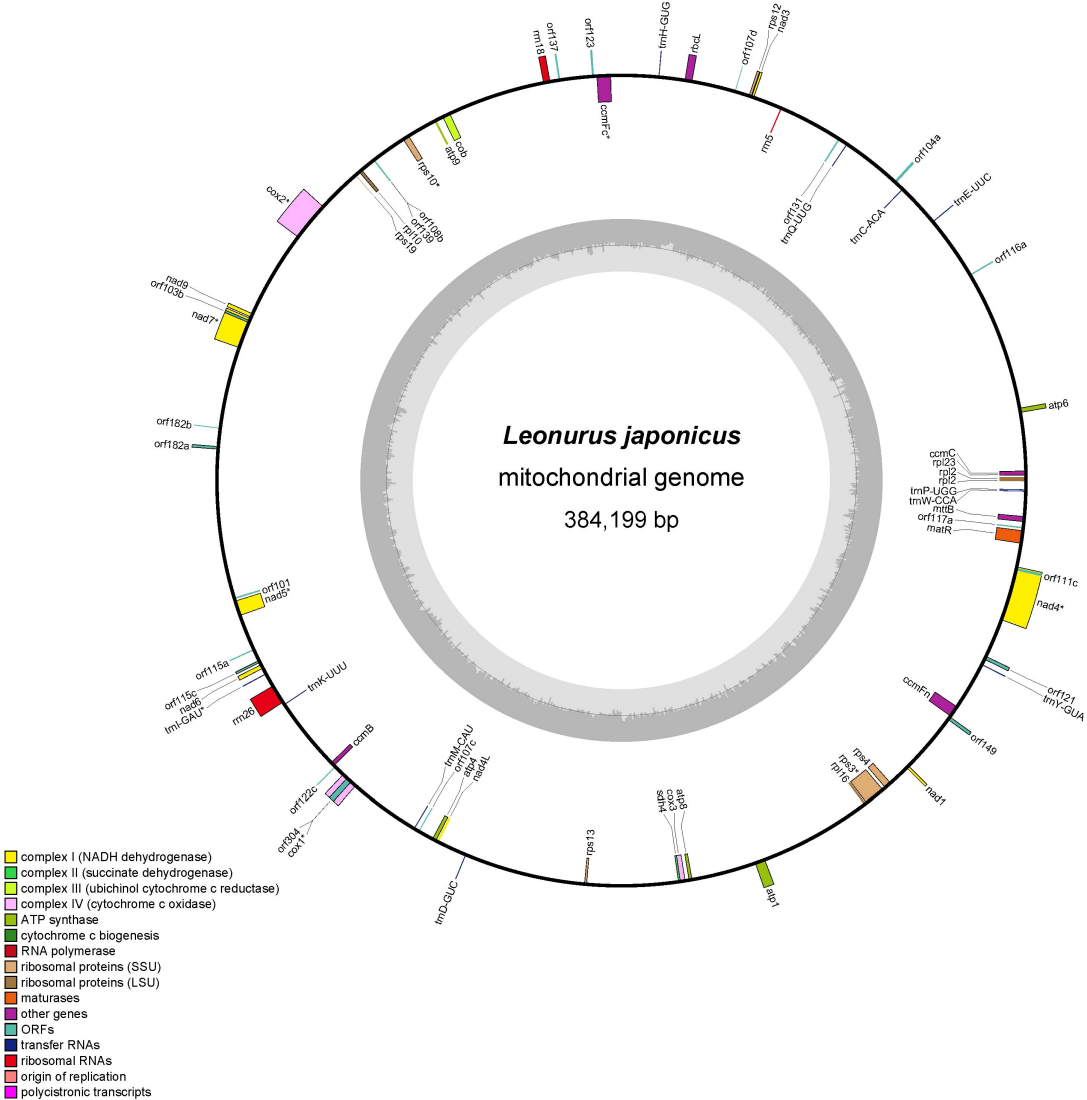
The putative circular mitogenome maps of *L. japonicus*. Different color blocks represent various functional gene groups.

In this study, the *L. japonicus* mitogenome was assembled into a circular molecule with a total length of 384,199 bp (**Figure 1**). The overall GC content of the assembled mitogenome is 45.1% (**Table 1**). Annotation revealed 35 unique mitochondrial protein-coding genes, comprising 24 core genes and 11 non-core genes, along with 11 tRNA genes and 3 rRNA genes (**Table 2**). The core gene set includes five ATP synthase genes (*atp*1, *atp*4, *atp*6, *atp*8, *atp*9), nine NADH dehydrogenase genes (*nad*1, *nad*2, *nad*3, *nad*4, *nad*4L, *nad*5, *nad*6, *nad*7, *nad*9), one cytochrome *b* gene (*cob*), four cytochrome *c* biogenesis genes (*ccm*B, *ccm*C, *ccm*FC, *ccm*FN), three cytochrome *c* oxidase genes (*cox*1, *cox*2, *cox*3), one membrane transport protein gene (*mtt*B), and one maturase gene (*mat*R). The non-core genes include four large ribosomal subunit genes (*rpl*2, *rpl*10, *rpl*16, *rpl*23), six small ribosomal subunit genes (*rps*3, *rps*4, *rps*10, *rps*12, *rps*13, *rps*19), and one succinate dehydrogenase gene (*sdh*4).

**Table 1:** Basic information of the mitogenome of *L. japonicus*.

Contigs	Type	Length	GC content
Chromosome 1	circular	384,199 bp	45.1%

**Table 2:** Gene content of the *L. japonicus* mitogenome.

Group of Genes	Name of Genes
ATP synthase	*atp*1, *atp*4, *atp*6, *atp*8, *atp*9
NADH dehydrogenase	*nad*1, *nad*2****, *nad*3, *nad*4***, *nad*4L, *nad*5*, *nad*6, *nad*7***, *nad*9
Cytochrome b	*cob*
Cytochrome *c* biogenesis	*ccm*B, *ccm*C, *ccm*FC*, ccmFN
Cytochrome *c* oxidase	*cox*1*, *cox*2*, *cox*3
Maturases	*mat*R
Protein transport subunit	*mtt*B
Ribosomal protein large subunit	*rpl*2, *rpl*10, *rpl*16, *rpl*23
Ribosomal protein small subunit	*rps*3*, *rps*4, *rps*10*, *rps*12, *rps*13, *rps*19
Succinate dehydrogenase	*sdh*4
Ribosome RNA	*rrn*5, *rrn*26, *rrn*18
Transfer RNA	*trn*C-ACA, *trn*D-GUC, *trn*E-UUC, *trn*H-GUG, *trn*I-GAU*, *trn*K-UUU, *trn*M-CAU, *trn*P-UGG, *trn*Q-UUG, *trn*W-CCA, *trn*Y-GUA

The asterisks (*) indicate the number of introns within the genes: one asterisk (*) represents one intron and three asterisks (***) represent three introns.

### Selective pressure analysis

To investigate evolutionary constraints acting on mitochondrial genes, we conducted Ka/Ks ratio analysis of conserved protein-coding genes between *L. japonicus* and its related species. The results revealed that the majority of genes were subject to purifying selection (Ka/Ks < 1), indicating strong functional conservation during evolution. For example, genes such as *atp*6 and *cox*1 consistently exhibited low Ka/Ks ratios (< 0.3) across multiple species. Notably, several genes showed signs of positive selection (Ka/Ks > 1), including *atp*4 in *Salvia miltiorrhiza* (Ka/Ks = 2.00885) and *ccm*B in *Ajuga ciliata* (Ka/Ks = 1.18703), suggesting that these genes may have undergone adaptive evolution associated with specific environmental conditions or lineage-specific metabolic processes (**Supplementary Table S1**).

### Analysis of repeat elements

**Figure 2:**
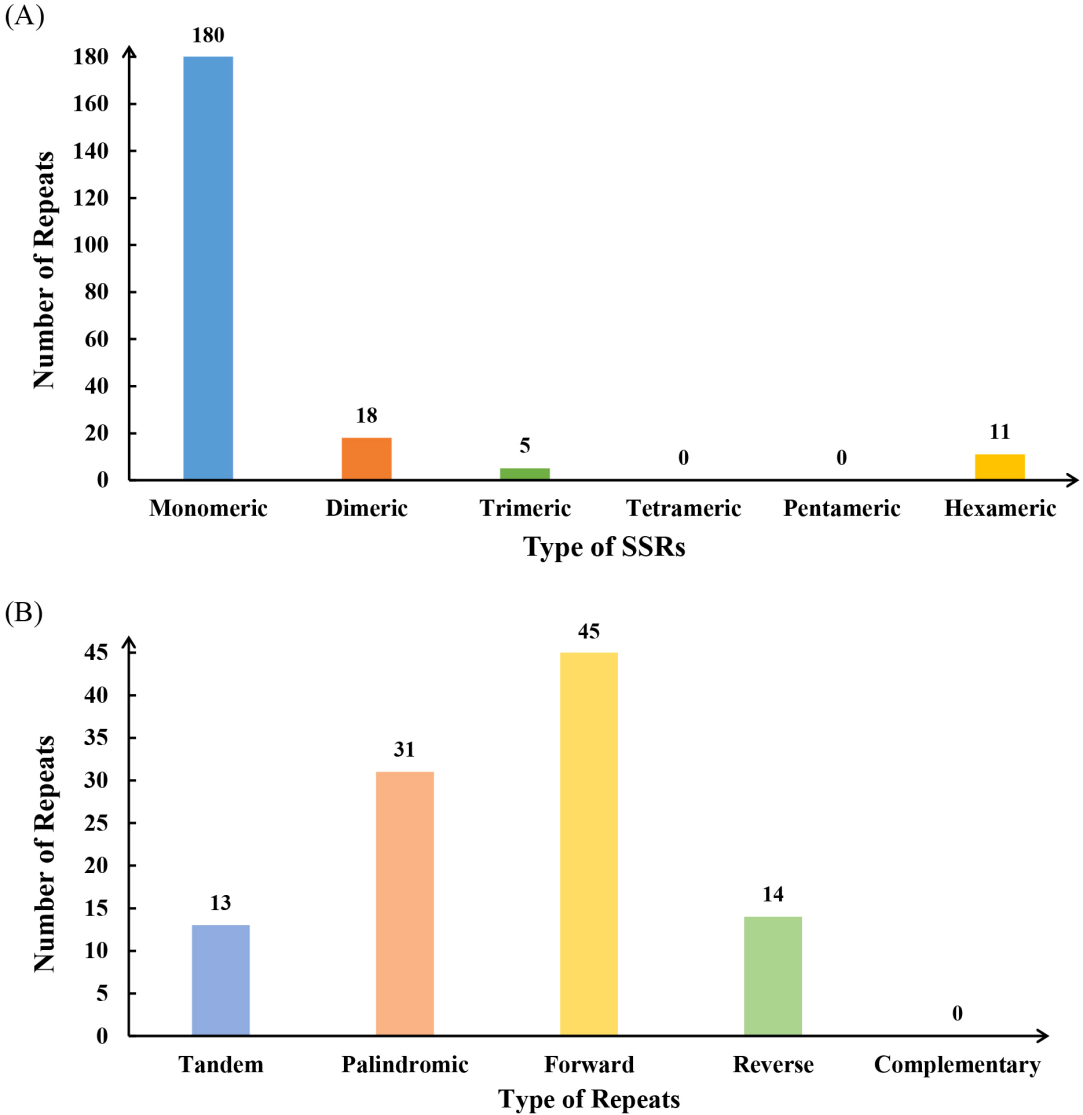
The simple sequence repeats (SSRs) and dispersed repeats identified in the mitogenome of *L. japonicus*. **(A)** The SSRs identified in the *L. japonicus* mitogenome, with each column representing different nucleotide repeat units displayed in various colors. **(B)** Dispersed repeats (≥ 30 bp) identified in the *L. japonicus* mitogenome.

Repetitive sequence analysis of the *L. japonicus* mitogenome identified a total of 241 SSRs, with mononucleotide and dinucleotide motifs comprising 92.52% of all SSRs. Among the 180 mononucleotide repeats, adenine (A) motifs were the most prevalent, accounting for 47.78% (n = 86) (**Supplementary Table S2**). Additionally, 11 hexanucleotide SSRs were detected (**Figure 2A**). The mitogenome also contains 13 tandem repeats (**Figure 2B**, **Supplementary Table S3**), a type of repeat sequence commonly observed in both eukaryotic and prokaryotic genomes. Analysis of dispersed repeats revealed 90 pairs of sequences with lengths ≥30 bp, including 45 forward repeats, 31 palindromic repeats, and 14 reverse repeats. The longest palindromic and forward repeats measured 3,004 bp and 4,625 bp, respectively (**Supplementary Table S4**).

### Codon usage analysis of PCGs

**Figure 3:**
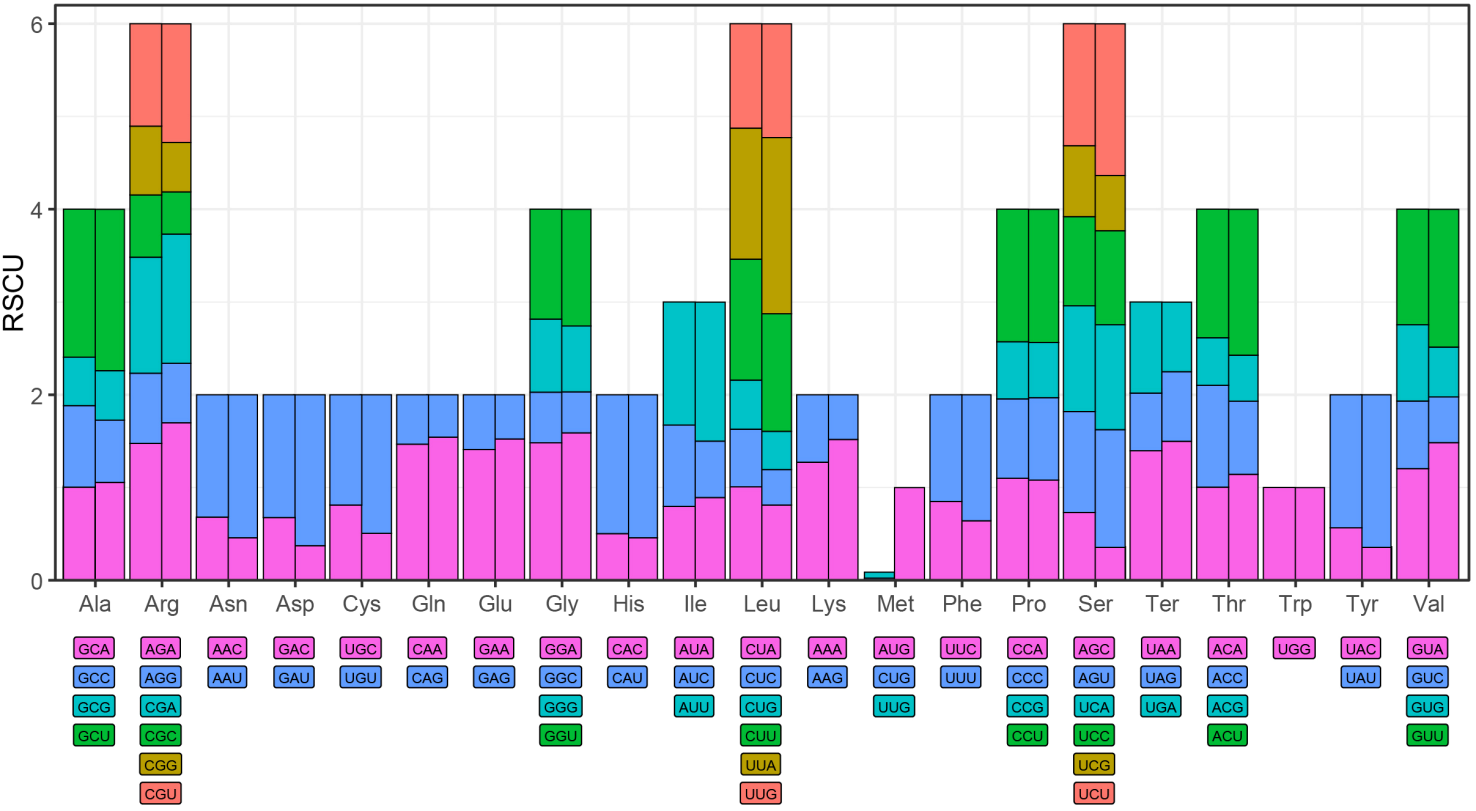
Relative synonymous codon usage (RSCU) analysis of mitochondrial and plastid protein-coding genes in *L. japonicus.* Left: mitochondrial; Right: plastid.

Codon usage bias was examined across 35 unique PCGs in the *L. japonicus* mitogenome and compared to that of its plastome. The RSCU values for each codon in both organellar genomes are summarized in **Supplementary Table S5**. Codons with RSCU values greater than 1 were considered preferentially utilized. As illustrated in **Figure 3**, both genomes exhibited similar amino‐acid codon usage bias, except for methionine. For instance, alanine’s GCU codon had RSCU values of 1.60 in the mitogenome versus 1.74 in the plastome, and arginine’s AGA codon rose from 1.47 to 1.70, indicating more pronounced bias in the plastome. GCU showed the highest RSCU value in both genomes, reflecting a pronounced usage preference. Termination codon usage also paralleled this trend: UAA was favored over UGA and UAG, with RSCU values of 1.40 in the mitogenome and 1.50 in the plastome, suggesting differing pressures on translation termination codon selection between the two organelles.

### Phylogenomic inference of *L. japonicus* using conserved mitochondrial protein-coding genes

**Figure 4:**
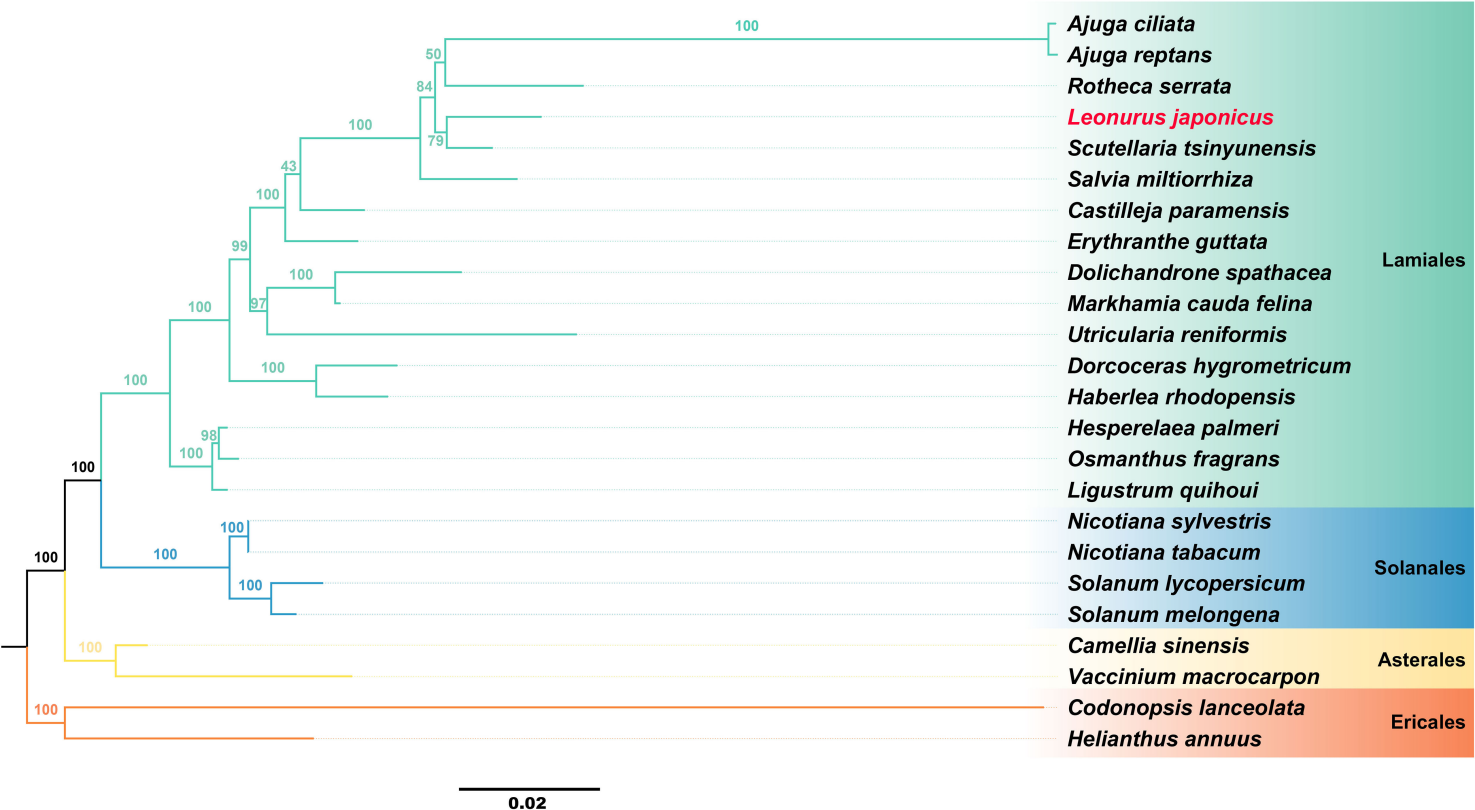
Phylogenetic analyses of *L. japonicus* based on mitogenome. The plants in the diagram belong to the order Lamiales. Different orders are represented by different colors, with *L. japonicus* represented in red.

To better elucidate the evolutionary relationships of *L. japonicus*, a phylogenetic analysis was conducted based on its mitogenome, along with those of 23 other published plant species. The results confirmed that *L. japonicus* is a member of the Lamiaceae family and is most closely related to *Scutellaria tsinyunensis*, *Rotheca serrata*, *Ajuga reptans*, and *A. ciliata* (**Figure 4**). This mitogenomic framework provides a robust basis for future phylogenetic and comparative studies of *L. japonicus*. A complete list of the analyzed species and their corresponding NCBI accession numbers is provided in **Supplementary Table S6**.

### Comparative mitogenome synteny and intracellular gene transfer in *L. japonicus*

**Figure 5:**
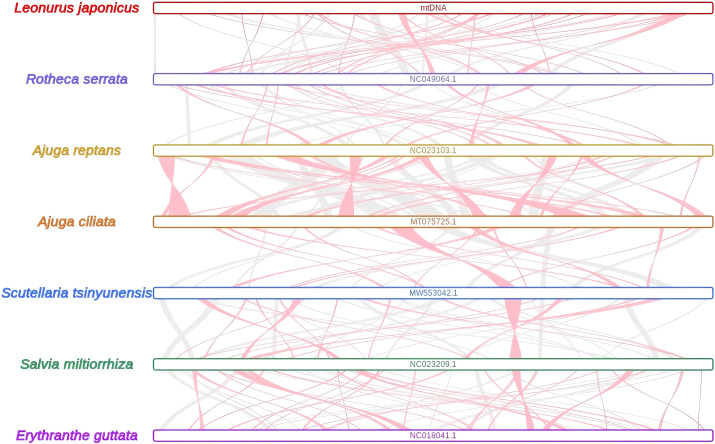
The collinear blocks among mitogenomes. Bars indicated the mitogenomes, and the ribbons showed the homologous sequences (> 500 bp) between the adjacent species. The red areas and gray areas indicate collinear blocks with inconsistent and consistent arrangement orders, respectively.

Extensive homologous collinear blocks were identified between *L. japonicus* and several closely related species within Lamiales, including *Rotheca serrata*, *Ajuga reptans*, *A. ciliata*, *Scutellaria tsinyunensis*, *Salvia miltiorrhiza*, and *Erythranthe guttata* (**Figure 5**, **Supplementary Table S7**). These regions demonstrate high levels of sequence similarity and conserved gene order, though some regions in *L. japonicus* lack homologous counterparts, indicating unique structural features. The synteny analysis also revealed frequent recombination and inversion events in the mitogenomes of these taxa.

**Figure 6:**
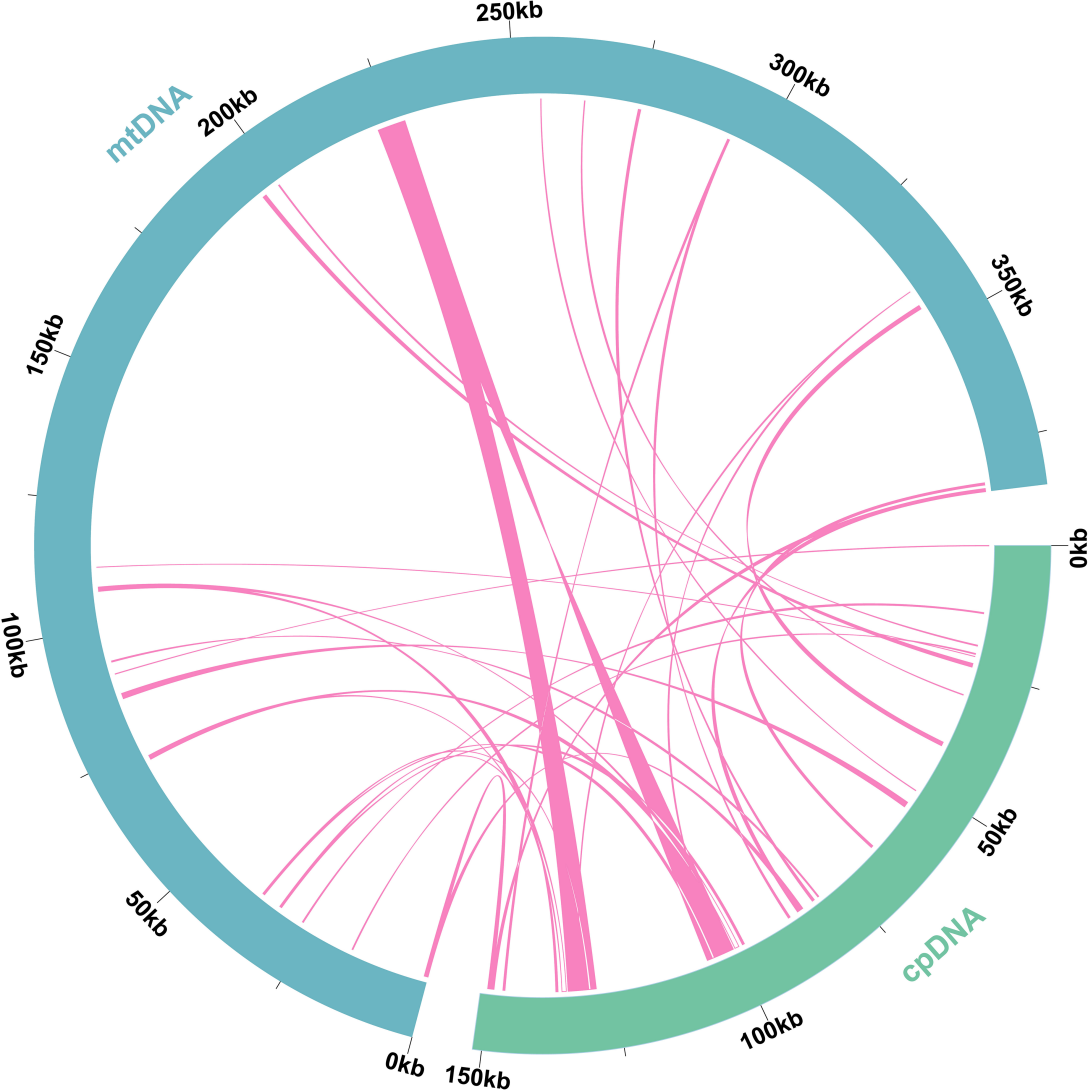
Schematic representation of homologous sequences between the plastome and mitogenome in *L. japonicus*. Blue arcs represent mitogenome, green arcs represent the plastome, and the lines between arcs correspond to homologous genome segments.

To identify MTPTs, we assembled the plastome of *L. japonicus*, which measured 151,630 bp. Based on sequence similarity, a total of 31 MTPTs were identified, with a combined length of 24,818 bp—representing 6.46% of the mitogenome and 16.37% of the plastome (**Figure 6**, **Supplementary Table S8**). Three fragments exceeded 1,000 bp in length, with MTPT1 and MTPT2 reaching up to 5,609 bp. Gene annotation revealed two complete genes (*rrn*23 and *trn*P-UGG) among these regions.

**Figure 7:**
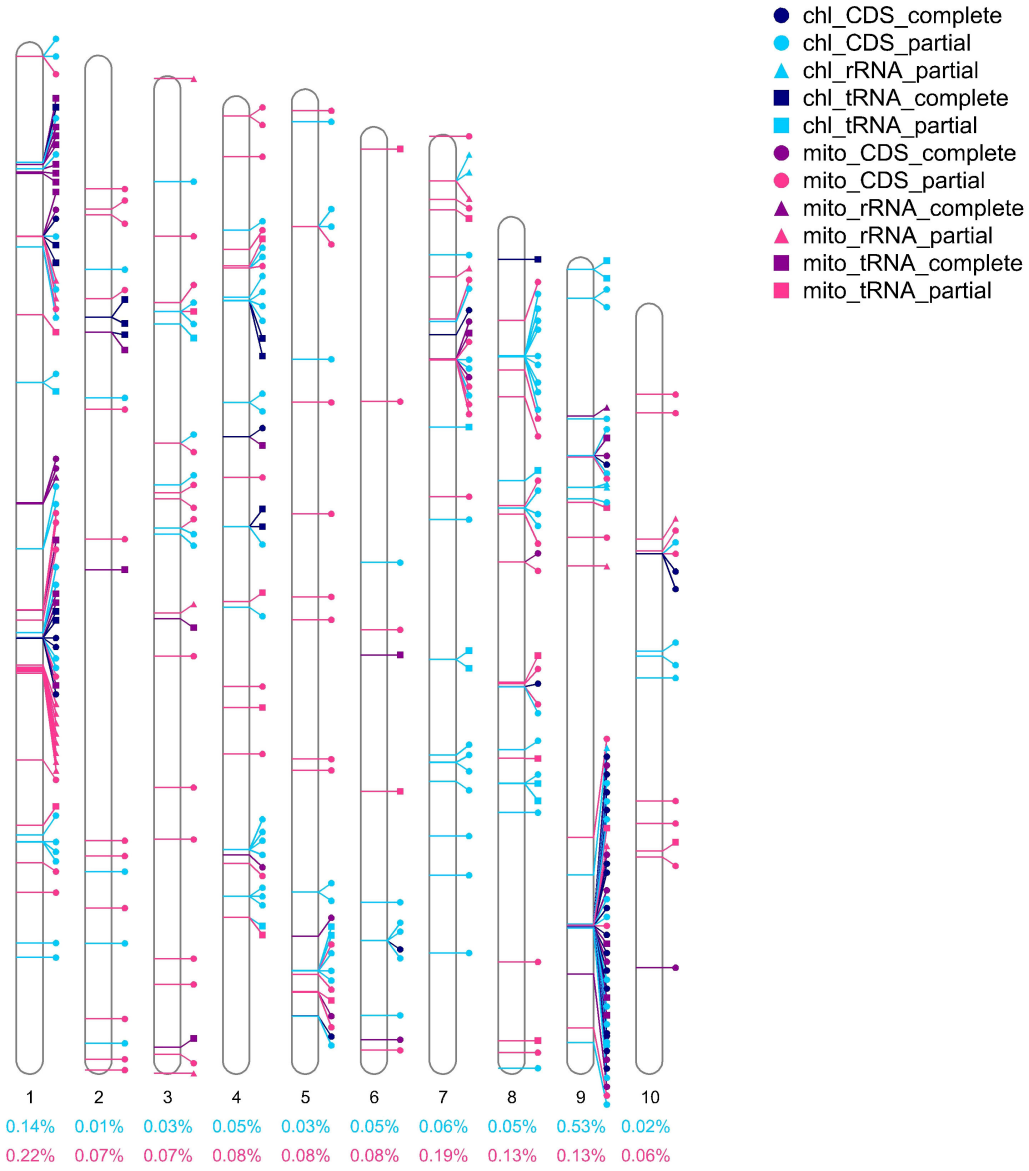
Homologous genes from *L. japonicus* organelle genomes on nuclear chromosomes are labeled. Icons near the chromosomes indicate the positions of genes from organelles (plastids and mitochondria, distinguished by purple and blue colors) on chromosomes. Genes are divided into CDS, rRNA, and tRNA types and are distinguished by three shapes (circle, square, and triangle). Complete and partial genes were indicated with dark and light colors, respectively. The percentage numbers below the chromosomes represent the percentage of the length of the homologous segment on that chromosome, with blue numbers representing the plastid homologous segment and pink numbers representing the mitochondrial homologous segment.

In addition, a substantial number of organelle-derived homologous fragments and genes were detected in the nuclear genome of *L. japonicus*. In total, 994,321 bp of nuclear DNA showed homology to organellar genomes—comprising 457,425 bp from plastid-derived sequences and 536,896 bp from mitochondrial-derived sequences—within a nuclear genome totaling 485,856,522 bp (**Supplementary Table S9**). The proportion of homologous fragments on individual chromosomes was relatively low; for example, chromosome 9 contained the highest proportion of plastid-derived sequences, accounting for only 0.53% of its length (**Figure 7**). A total of 341 organellar genes were mapped to the nuclear genome, including 177 plastid genes (40 complete) and 164 mitochondrial genes (43 complete) (**Supplementary Table S10**). Plastid-to-nucleus homologous sequences accounted for 0.09% of the nuclear genome, and mitochondria-to-nucleus sequences accounted for 0.11% (**Supplementary Table S9**). Among complete transferred plastid genes, *infA* (four copies) and *rpl*2 (five copies) were the most frequently observed (**Supplementary Table S11**).

### The prediction of RNA editing events

**Figure 8:**
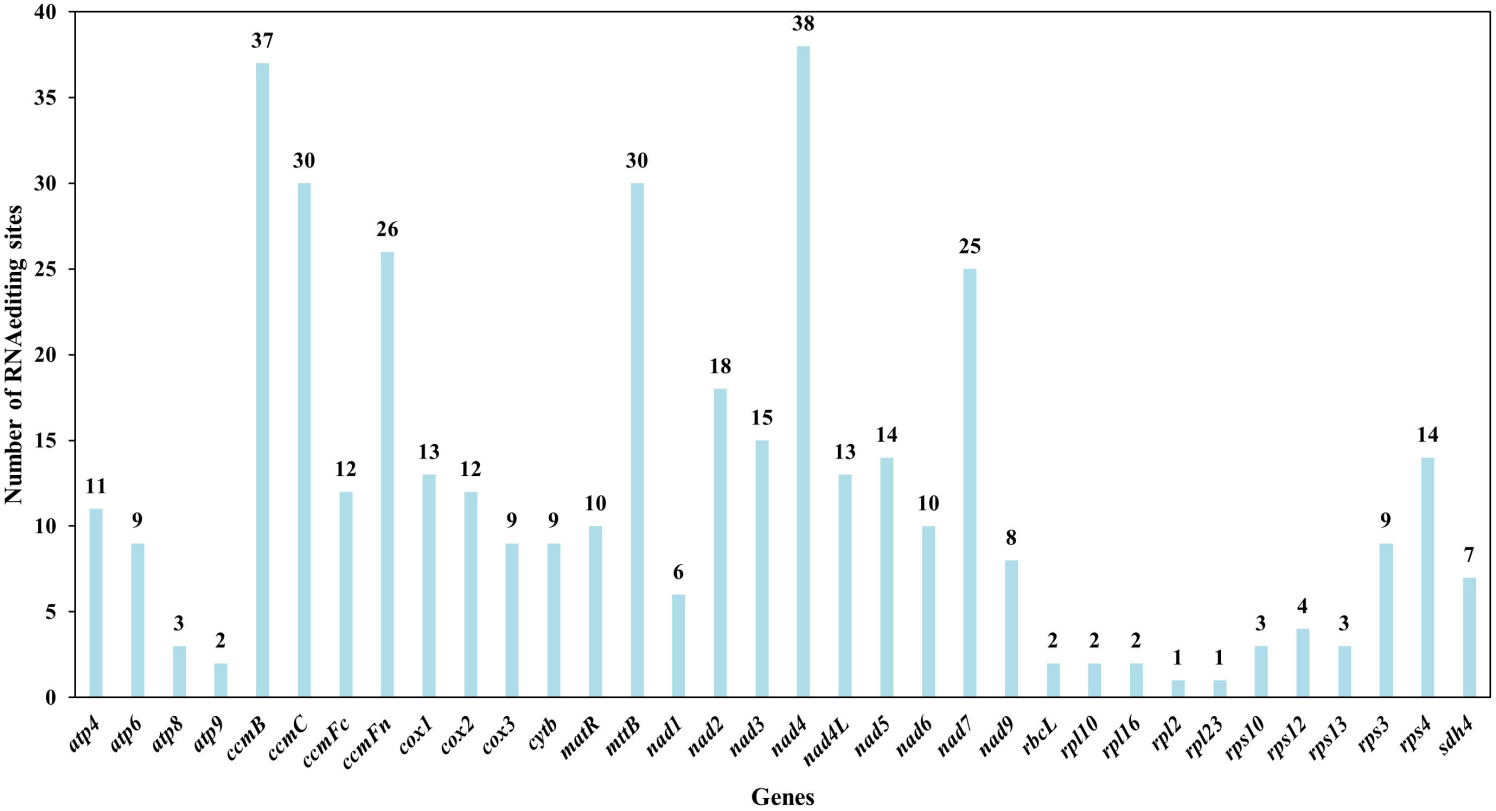
Characteristics of the RNA editing sites identified in protein-coding genes (PCGs) of *L. japonicus* mitogenome. Number of RNA editing sites predicted by individual PCGs using Deepred-mt. The x-axis shows the gene names, and the y-axis shows the number of edited sites.

RNA editing events were predicted across 35 unique PCGs in the *L. japonicus* mitogenome using Deepred-mt, with a confidence threshold of 0.9. A total of 408 C-to-U editing sites were identified (**Figure 8**, **Supplementary Table S12**). These editing events were found to alter protein-coding potential, including creating new start and stop codons. Specifically, a novel start codon was predicted in *cox*2 and a novel stop codon in *rps*10. Among all genes, *nad*4 harbored the highest number of predicted RNA editing sites (38 sites), followed by *ccm*B with 37 edits, suggesting that these genes may undergo extensive post-transcriptional modification.

## Discussion

### Size and genetic composition properties of the mitogenome

By using long-read sequencing, we assembled a high-quality, full-length mitogenome of *L. japonicus*, totaling 384,199 bp. This genome size is larger than that of other Lamiaceae species, such as *Pogostemon heyneanus* (380,655 bp), *Scutellaria barbata* (372,525 bp), *Lavandula angustifolia* (355,345 bp), and *Ajuga reptans* (352,069 bp), but smaller than those of *Salvia miltiorrhiza* (499,236 bp), *Platostoma chinense* (494,599 bp), and *Rotheca serrata* (482,114 bp). The GC content of the *L. japonicus* mitogenome is 45.1%, comparable to that of other Lamiaceae species, ranging from 44.21% to 45.54%. This conserved GC content supports the notion that GC levels in plant mitogenomes have remained relatively stable throughout angiosperm evolution.

In terms of gene composition, the *L. japonicus* mitogenome contains a typical complement of mitochondrial genes, including 35 protein-coding genes involved in oxidative phosphorylation—such as subunits of NADH dehydrogenase, ATP synthase, cytochrome c oxidase, and cytochrome b—as well as three rRNA genes (*rrn*5, *rrn*18, *rrn*26) and a complete set of tRNAs representing most amino acids (Yang et al., 2023; Sun et al., 2024). Comparative analysis indicates that, although the core gene set is generally conserved across Lamiaceae, variations in gene content and the presence of additional open reading frames (ORFs) may account for observed genome size differences. These variations likely result from gene duplications, losses, and rearrangements, which not only shape genome architecture but also reflect lineage-specific evolutionary processes (Crombach and Hogeweg, 2007; Katju and Bergthorsson, 2013).

Furthermore, selective pressure analysis among *L. japonicus* and its close relatives (*Scutellaria tsinyunensis*, *Rotheca serrata*, *Ajuga reptans*, *A. ciliata, and Salvia miltiorrhiza*) revealed that most mitochondrial respiratory genes (*nad*1*–nad*7, *cox*1*–cox*3, *atp*6, *atp*9) are under strong purifying selection, indicating functional conservation essential for energy metabolism. This suggests that adaptation to fluctuating light and temperature conditions may be mediated through the modulation of NADH dehydrogenase and ATP synthase activities (Heidarvand et al., 2017; Yokochi et al., 2021). In contrast, *ccm*B and *nad*4 exhibited signs of positive selection in certain species, potentially reflecting adaptive responses to oxidative stress or enhanced metabolic efficiency. In particular, the positive selection of *ccm*B may contribute to cytochrome c stabilization under high oxidative conditions (Sanders et al., 2010).

### Repeated sequences and extensive homologous recombination in the mitogenome

Repetitive sequences are widely distributed in plant mitogenomes and are known to contribute to genome expansion, regulation of gene expression, and the evolution of complex traits (Mehrotra and Goyal, 2014; Wynn and Christensen, 2019; Xiong et al., 2022). In the *L. japonicus* mitogenome, we identified a total of 241 SSRs, providing a valuable set of potential molecular markers for species identification and genetic diversity studies in *Leonurus* germplasm. The prevalence of dispersed repeats suggests their likely involvement in genome structural variation and regulatory functions (Gualberto and Newton, 2017; Wynn and Christensen, 2019) Repetitive elements in plant mitogenomes are also closely associated with homologous recombination, a key mechanism for genomic rearrangements and evolutionary innovation (Knoop, 2012; Christensen, 2013). In our synteny analysis, *L. japonicus* showed numerous homologous collinear blocks with other Lamiales species; however, these regions were relatively short, and several sequence gaps—i.e., regions lacking detectable homology—were identified. These unique, species-specific segments may represent lineage-specific insertions or recombination-derived sequences. Moreover, extensive recombination was detected within the mitogenome, as indicated by frequent inversion and rearrangement events among conserved regions (**Figure 5**). The observed high rate of structural reshuffling among Lamiales mitogenomes suggests that mitogenome organization in this clade is relatively dynamic and poorly conserved. This pattern is consistent with previous findings that repeated sequence-mediated recombination contributes to structural plasticity in plant mitogenomes and may play an adaptive role under environmental stress conditions (Hernández-Hernández et al., 2014; Copetti et al., 2017).

### Organelle-to-nuclear gene transfer in *L. japonicus*: evolutionary implications and prospects for genetic improvement

Due to their dynamic structural architecture and recombinational flexibility, plant mitogenomes are particularly permissive to the integration of foreign DNA (Wu et al., 2022). A well-documented example of this phenomenon is the incorporation of plastid-derived DNA fragments into the mitogenome, resulting in MTPTs (Wang et al., 2007; Alverson et al., 2011; Gao et al., 2020). In *L. japonicus*, we identified 31 MTPTs spanning a total of 24,818 bp, which constitutes 16.37% of its plastome length—a relatively high proportion compared to most angiosperms and gymnosperms. A similar pattern of plastid-to-mitochondrion transfer has also been reported in *Selenicereus monacanthus* (Lu et al., 2023). These transferred fragments often include partial or complete genes, including those originally involved in photosynthesis (Alverson et al., 2011), although in *L. japonicus*, only two complete genes (*rrn*23 and *trn*P-UGG) were retained.

Beyond the interaction between organelles, gene transfer from mitochondria and plastids to the nuclear genome is a hallmark of endosymbiotic evolution and has contributed significantly to shaping modern plant genomes (Timmis et al., 2004). In *L. japonicus*, we identified a total of 994,321 bp of nuclear-integrated organellar homologs, including 457,425 bp from plastid origin and 536,896 bp from mitochondrial origin. These homologous sequences were distributed across all chromosomes, although in varying proportions, with chromosome 9 showing the highest density of plastid-derived fragments (**Figure 7**, **Supplementary Table S9**). Among 341 identified nuclear-localized organelle genes, 40 plastid and 43 mitochondrial genes were found to be complete (**Supplementary Table S10**), with multiple gene copies detected—e.g., five copies of *rpl*23 and eight of *trn*P-UGG—likely reflecting selective amplification to meet cellular demands (Galeota-Sprung et al., 2022; Zwonitzer et al., 2024).

Functionally, the integration of organelle-derived genes into the nuclear genome may confer regulatory advantages. For instance, transferred genes such as *atp*8, *rps*10, *rpl*23, *rrn*18, and *rrn*5 retain essential functions in mitochondrial translation and energy metabolism while benefiting from nuclear transcriptional regulation (Kleine et al., 2009; Wu et al., 2024). This relocation facilitates the coordination of gene expression with cellular energy demands and environmental signals. Under stress conditions, nuclear regulation allows rapid modulation of these transferred genes, enhancing organelle protection and ensuring cellular viability (Korpelainen, 2004; Drouin et al., 2008; Rogalski et al., 2008). Furthermore, these nuclear-integrated organelle genes often exhibit sequence divergence, undergo RNA editing, or acquire novel regulatory features that may lead to functional innovation (Lu and Li, 2024). In *L. japonicus*, for instance, transferred genes like *atp*8, *rrn*18, and *rpl*23 not only retain their core mitochondrial roles but also show increased copy numbers, potentially supporting elevated translational capacity under high metabolic demand. Overall, the extensive plastid-to-mitochondrion and organelle-to-nucleus gene transfer events observed in *L. japonicus* not only reflect its complex evolutionary history but also underscore the importance of intercompartmental genome interaction in shaping plant genomic plasticity, adaptation, and mitochondrial function (Wu et al., 2024).

Moreover, understanding and manipulating these gene transfer events could provide novel strategies for breeding. Targeted modification of transferred genes may enhance stress tolerance or metabolic efficiency. Additionally, the instability of organellar genomes—particularly under environmental stress—can influence nuclear genome behavior through retrograde signaling. For example, plastome instability has been shown to induce nuclear responses that mediate DNA repair and regulate cell cycle progression (Mahapatra et al., 2021), further linking organelle dynamics to whole-cell genome integrity and stress resilience.

### RNA editing events are prevalent in the PCGs of the mitogenome

RNA editing is a widespread post-transcriptional modification in higher plant organelles, predominantly involving C-to-U conversions that result in transcripts differing from the DNA template (Edera et al., 2018; Hao et al., 2021). This process often restores functional reading frames by introducing start codons or removing premature stop codons, thereby contributing to proper protein synthesis (Edera et al., 2018; Small et al., 2020). In *L. japonicus*, RNA editing was predicted in all 35 mitochondrial PCGs, resulting in 408 editing sites—each potentially altering the encoded amino acid and conferring novel structure or function (Møller et al., 2021). Notably, RNA editing generated new start and stop codons in *cox*2 and *rps*10, respectively (**Figure 8**, **Supplementary Table S12**). Such edits typically enhance the conservation and homology of gene products with those from other species, improving mitochondrial gene expression (Covello and Gray, 1989). Several functionally relevant edits were observed in respiratory genes. For example, *cox*2, encoding a subunit of cytochrome c oxidase, is subject to temperature-responsive RNA editing, influencing transcript maturation in wheat (Kurihara-Yonemoto and Handa, 2001). Similarly, *ccm*C edits shifted serine to hydrophobic residues such as leucine or phenylalanine, which may enhance membrane association or protein interactions (Giegé et al., 2008; Shevket et al., 2018).

RNA editing has broader implications for plant development and breeding. It can be harnessed for trait improvement, such as enhancing disease resistance or stress tolerance by manipulating editing sites (Chu and Wei, 2019). Furthermore, editing has been linked to CMS, a trait exploited in hybrid breeding. In CMS-T maize, for instance, editing-induced alterations in the mitochondrial *orf*355*-orf*77 gene disrupted mitochondrial function and caused pollen abortion (Schnable, 1998; Chase, 2007). Such associations highlight the critical role of RNA editing in mitochondrial regulation and its potential utility in crop improvement strategies.

## Conclusion

In this study, we assembled and annotated the complete mitogenome of *L. japonicus*, providing a comprehensive overview of its genetic structure and evolutionary features. The mitogenome, assembled as a circular molecule of 384,199 bp, encodes a typical set of mitochondrial protein-coding genes, tRNAs, and rRNAs, alongside numerous repetitive sequences contributing to genome plasticity. Extensive RNA editing was detected across protein-coding genes, suggesting widespread post-transcriptional modifications that may alter protein structure and function. Comparative analyses revealed evidence of MTPTs, organelle-to-nuclear gene transfer, and collinearity variation among related species, highlighting the evolutionary dynamism of the *L. japonicus* mitogenome. These findings not only deepen our understanding of mitogenome evolution in the Lamiaceae but also offer valuable genomic resources for molecular breeding, stress adaptation research, and the development of new medicinal applications.

## Data Availability

The mitochondrial and plastid genomes generated in the study are publicly available. These data are available at NCBI (https://www.ncbi.nlm.nih.gov/) under the GenBank: PV368446 (https://www.ncbi.nlm.nih.gov/nuccore/PV368446.1) and PP882658 (https://www.ncbi.nlm.nih.gov/nuccore/PP882658.1). The raw sequencing reads from the Illumina and Nanopore platforms have been deposited in NCBI with accession number PRJNA1120637 (https://www.ncbi.nlm.nih.gov/bioproject/PRJNA1120637).
